# Identification of homozygous missense variant in *SIX5* gene underlying recessive nonsyndromic hearing impairment

**DOI:** 10.1371/journal.pone.0268078

**Published:** 2022-06-16

**Authors:** Mohib Ullah Kakar, Muhammad Akram, Muhammad Zubair Mehboob, Muhammad Younus, Muhammad Bilal, Ahmed Waqas, Amina Nazir, Muhammad Shafi, Muhammad Umair, Sajjad Ahmad, Misbahuddin M. Rafeeq

**Affiliations:** 1 Faculty of Marine Sciences, Lasbela University of Agriculture, Water and Marine Sciences (LUAWMS), Uthal, Balochistan, Pakistan; 2 Department of Life Sciences, School of Science, University of Management and Technology (UMT), Lahore, Pakistan; 3 CAS Center for Excellence in Biotic Interaction, College of Life Sciences, University of Chinese Academy of Science, Beijing, China; 4 State Key Laboratory of Membrane Biology, Beijing Key Laboratory of Cardiometabolic Molecular Medicine, Institute of Molecular Medicine, Peking-Tsinghua Center for Life Sciences, PKU-IDG/McGovern Institute for Brain Research, Peking University, Beijing, China; 5 Department of Biochemistry, Faculty of Biological Sciences, Quaid-i-Azam University, Islamabad, Pakistan; 6 Division of Science and Technology, Department of Zoology, University of Education Lahore, Lahore, Pakistan; 7 Institute of Animal Science and Veterinary Medicine, Shandong Academy of Agricultural Sciences, Shandong Province, China; 8 Faculty of Veterinary and Animal Sciences, Lasbela University of Agriculture, Water and Marine Sciences (LUAWMS), Uthal, Balochistan, Pakistan; 9 Department of Pharmacology, Faculty of Medicine, Rabigh King Abdul Aziz University, Jeddah, KSA; National Institute of Dental Research: National Institute of Dental and Craniofacial Research, UNITED STATES

## Abstract

Hearing impairment (HI) is a heterogeneous condition that affects many individuals globally with different age groups. HI is a genetically and phenotypically heterogeneous disorder. Over the last several years, many genes/loci causing rare autosomal recessive and dominant forms of hearing impairments have been identified, involved in various aspects of ear development. In the current study, two affected individuals of a consanguineous family exhibiting autosomal recessive nonsyndromic hearing impairment (AR-NSHI) were clinically and genetically characterized. The single affected individual (IV-2) of the family was subjected to whole-exome sequencing (WES) accompanied by traditional Sanger sequencing. Clinical examinations using air conduction audiograms of both the affected individuals showed profound hearing loss across all frequencies. WES revealed a homozygous missense variant (c.44G>C) in the *SIX5* gene located on chromosome 19q13.32. We report the first case of autosomal recessive NSHI due to a biallelic missense variant in the *SIX5* gene. This report further supports the evidence that the *SIX5* variant might cause profound HI and supports its vital role in auditory function. Identification of novel candidate genes might help in application of future gene therapy strategies that may be implemented for NSHI, such as gene replacement using cDNA, gene silencing using RNA interference, and gene editing using the CRISPR/Cas9 system.

## 1. Introduction

Hearing impairment (HI) or deafness in humans is a complex disorder exhibiting a heterogeneous phenotypic and genetic landscape. HI is a heterogeneous condition that affects 1 in 500 newborns and over 360 million individuals globally [1]. Approximately 100 autosomal recessive genes of nonsyndromic hearing impairment (NSHI) have been identified so far. Because of significant locus variability and the rarity of several variants associated with autosomal recessive nonsyndromic hearing impairment (AR-NSHI), candidate genes of HI are often found within only one family [1]. Pleiotropy (alterations in these genes) plays a significant role in a disorder such as deafness, which might give rise to autosomal dominant or autosomal recessive, nonsyndromic and severe syndromic forms of HI. Additionally, more than 115 loci have been mapped for nonsyndromic HI, segregating in either dominant (DFNA) or a recessive (DFNB) fashion [2, 3] http://hereditaryhearingloss.org/) (OMIM; S1 Table).

Clinically, Branchio-Oto-Renal syndrome (BOR MIM#113650) is a heterogeneous autosomal dominantly inherited disorder having phenotypes such as pits, branchial arch defects, renal defects, and HI. BOR has a high prevalence, variable expressivity, and clinical heterogeneity. The syndrome has a prevalence of 0.1/4000 and accounts for ∼2% of profound HI [4, 5]. In BOR patients, the bronchial arch defects include abnormal pinnae, pits, and cervical fistulas, while, HI can be sensorineural, conductive, or often associated with preauricular pits or tags [6]. The heterozygous variants of *SIX1*, *EYA1*, and *SIX5* genes are responsible for BOR syndrome [7, 8]. Heterozygous missense variants in the *SIX5* (MIM 600963) gene have been reported by one group in patients with BOR syndrome [7]. Heimler and Lieber [9] investigated 16 individuals in a large, four-generation kindred exhibiting manifestations of BOR syndrome. Out of 16 individuals, only 4 presented abnormalities in all three processes, while 7 presented a branchial arch and/or hearing defects without renal abnormalities [9].

A consanguineous family was investigated in the current study with two affected individuals showing AR-NSHI phenotypes, revealing a biallelic missense variant in the *SIX5* gene, located on chromosome 19q13.32. Thus, supporting the involvement of *SIX5* gene variants in the etiology of NSHI.

## 2. Materials and methods

### 2.1 Research subjects

The current study is based on genetic and clinical findings of a consanguineous family, residing in a remote area near the Pakistan-Afghanistan border. The institutional review board (IRB) Taif University [Researchers Supporting Project Number: TURSP-2020/140)], approved the research study. The research study was performed in accordance with the declaration of Helsinki protocols. The current research study is based on genetic and clinical findings of a consanguineous family, residing in a remote area near the Pakistan-Afghanistan border. All the participating members obtained a signed informed consent form to conduct the research studies and data publication.

### 2.2 Audiogram analysis

Standardized audiometric research devices were used for this analysis in an audiological clinic. The systems comply with the ISO 8253–1 and 8253–3 specifications. Pure-tone audiometry and air conduction sound levels for both ears were calculated individually for each patient. The frequencies examined were 125, 250; 500; 750; 1000; 1,500; 2,000; 4,000; 6,000; and 8,000 Hz and HL (range:– 20–120 dB). A customized Matlab 2008 package (MathWorks, MA, USA) was used to prepare the details [10].

### 2.3 Extraction of genomic DNA

**Fig 1:**
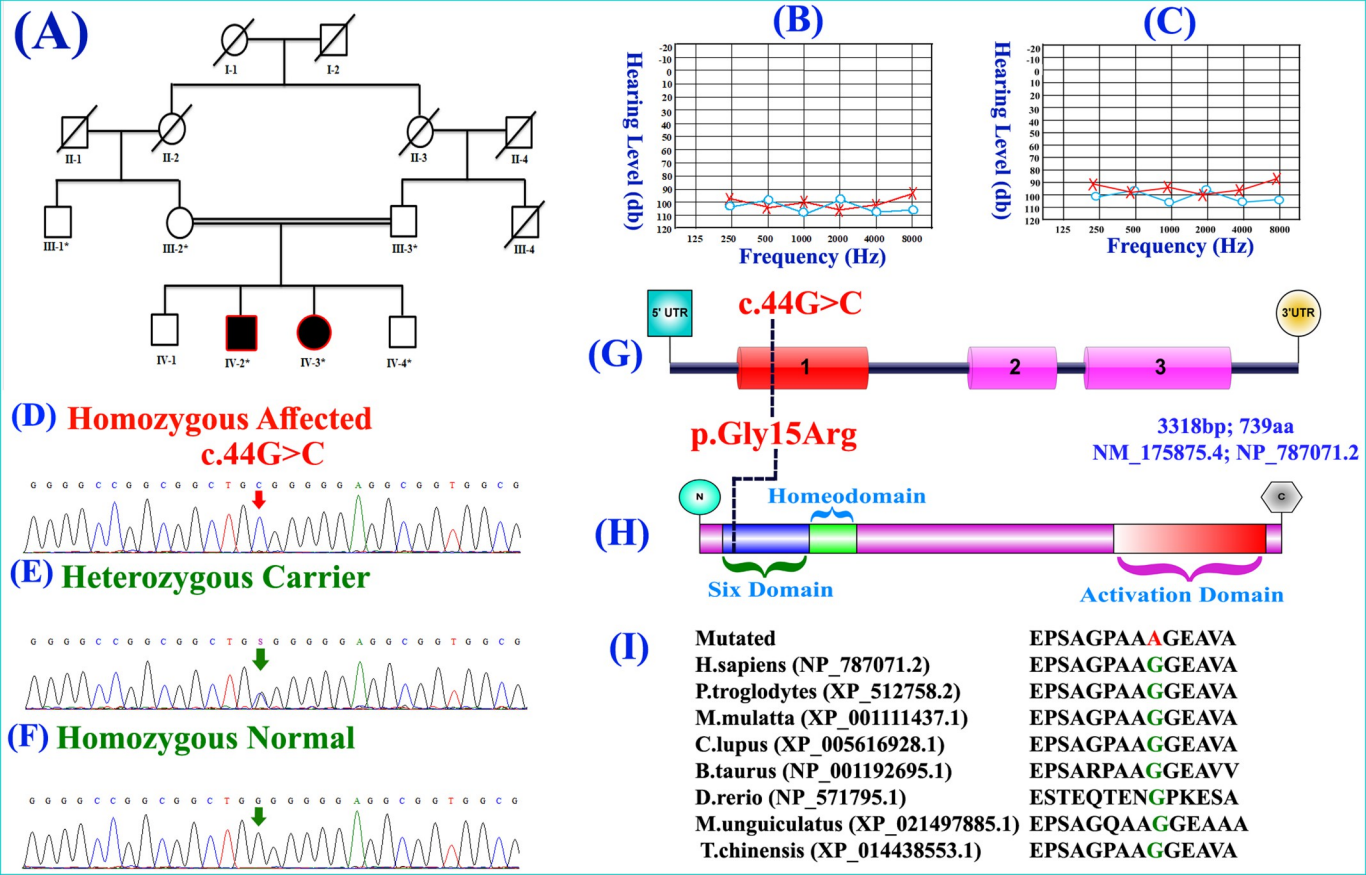
(**A**) Autosomal recessive inheritance pattern family pedigree. White circles and squares represent unaffected males and females, filled (black) circles and squares represent affected individuals, while crossed lines symbols are the deceased individuals. The consanguineous union is represented by double lines between symbols. Individuals with asterisks are the ones available for the present study. (**B, C**) Air conduction audiograms of affected individuals IV-2, and IV-3. Pure-tone audiometry was conducted between 250 and 8,000 Hz and red crosses, right ear; blue circles, left ear. Age at the time of audiometry (IV-2, 18 years; IV-3, 23 years). Individuals with the impairment have profound hearing loss at all frequencies. (**D**) Sanger sequencing electrograms showing co-segregation of the pathogenic missense variant (c.44G>C) identified in *SIX5* gene in homozygous affected, (**E**) heterozygous carrier and (**F**) homozygous normal individuals of the family. **(G, H)** Schematic representation of *SIX5* gene and protein. The text in red denotes the variant discovered in this analysis, which is located in exon 1 and the SIX domain (SD). The exons and introns have not been drawn to proportion. **(I)** Partial amino acid sequence of the SIX5 protein showing substitution of Glycine amino acid into Arginine amino acid and conservation of Glycine amino acid across different species.

Peripheral blood samples were collected from seven people, five unaffected (III-1, III-2, III-3, IV-1, IV-4) and two affected (IV-2, IV-3) in EDTA with vacutainer sets (BD, Franklin Lakes, NJ, USA) (Fig 1A). The Gene-Elute Blood Genomic DNA kit (Sigma-Aldrich, St Louis, MO, USA) was used to obtain the genomic DNA from the entire blood. The Nanodrop-1000 spectrophotometer (Thermal Scientific, Wilmington, St. Louis, MA, USA) was used for the quantification of the isolated DNA.

### 2.4 Whole Exome Sequencing (WES)

DNA of a single affected individual (IV-2) was analyzed by WES on the HiSeq2000 platform (Illumina, Inc., San Diego, CA). Sure-Select XT Human All Exon 50 Mb kit (version 5; Agilent Technologies, CA) was employed to perform the exomes enrichment while, Burrows-Wheeler Aligner (BWA v 0.7.5) was employed to align all the reads against the human assembly hg19 (GRCh37). However, Software Asset Management (SAM) tools (v0.1.18) [11], PINDEL (v0.2.4t) [12], and Exome Depth (v1.0.0) [13], were used for variant calling. Subsequently, all the variants obtained after filtering were then analyzed and subjected to Sanger sequencing for segregation within the family.

### 2.5 Identification of variants

Considering autosomal recessive inheritance mode of the phenotype and consanguineous marriage in the family, rare homozygous or compound heterozygous variants in different genes associated with syndromic and nonsyndromic profound deafness were filtered (List obtained from OMIM; S1 Table). A signed informed consent form to perform the study and publication of data was obtained from all the participating members.

#### 2.5.1 Sanger sequencing of candidate variants

Exon Primer (http://ihg.gsf.de) and Primer 3 software (http://frodo.wi.mit.edu/primer3/) were employed to design the primers (Sequences will be provided on request) for amplification of the 286-bp fragment flanking the missense variant (c.44G>C) in the *SIX5* gene. PCR-amplified DNA purification and Sanger sequencing were conducted as described previously [14].

#### 2.5.2 Bioinformatics analysis

Exome Aggregation Consortium ExAC (http://exac.broadinstitute.org/), Exome Variant Server (EVS), 1000 Genomes (http://www.1000genomes.org), in-house 165 exomes and genome aggregation database (gnomAD) (V2.1.1, MacArthur) were used to further cross-check the frequency of selected variants. Missense variants were predicted using FATHMM, SIFT, PrimateAI and Varsome. Tool used for conservation of variants across different species was CLC sequence viewer (8.0 QIAGEN Aarhus).

#### 2.5.3 Three-dimensional modeling

The homeodomain helical structure of the protein was predicted by employing the TMHMM server v. 2.0 [15]. The 3D model of SIX5 was built using the I-TASSER server by ab initio/threading method [16]. The PROCHECK software was used to assess the stereochemical consistency of protein structure [17].

## 3. Results

### 3.1 Clinical description

The affected members from an early age were suffering from HI, and as a result, they also developed speech difficulties. The two affected individuals, IV-2 (18 years), IV-3 (23 years), suffered profound HI, as shown in the audiograms (Fig 1B and 1C). Both the affected individuals showed severe (71–95 dB) or profound (> 95 dB) HI across all the frequencies, while apparently no signs of vestibular dysfunction were observed. However, abnormal pinnae (Grade 1), ear pits, and/or cysts were observed in the affected individuals and their parents, the most common branchial arch defects observed in BOR. The family members with HI were medically assessed, and neither severe vestibular dysfunction nor any vertigo episodes were found.

In the current family, parents did not reveal a history of hearing impairment, neurological, skeletal, and cardiovascular disorder. The affected individuals did not reveal any renal anomalies (hypoplasia, collecting system duplication, cystic dysplasia, agenesis and hydronephrosis) reported in several Branchio-oto-renal syndrome (BOR) cases.

### 3.2 Whole Exome Sequencing (WES)

WES of DNA of IV-2 was done as described previously [18, 19]. Obtained variants were filtered and validated according to MAF>0.001 in dbSNP by 1000 genome Project, 165 in-house exome database (Pakistani population), Exome Aggregation Consortium (ExAC), and gnomAD (http://gnomad.broadinstitute.org/). Disease causing variants (both homozygous and heterozygous) were screened followed by a systematic filtering process, leading to exploration of a homozygous missense variant (c.44G>C) existing in exon 1 of the *SIX5* gene (NM_175875.4; NP_787071.2). The variant identified here was presented in the heterozygous state with minor 0.00002398 allele frequency in the Genome Aggregation Consortium (gnomAD), while it was absent in the ExAC, 1000 Genome. The identified variant was screened in 250 controls and 300 exoms from Pakistani population. Pathogenicity of the missense variant was predicted using different online tools. The variant c.44G>C is classified as variant of uncertain significance (VUS) [PM2: GnomAD exomes allele count = 1 is less than 5 for gene SIX5 (good gnomAD exomes coverage = 23.1). Variant not found in gnomAD database (good gnomAD genomes coverage = 29.4)]. WES and Sanger sequencing (Fig 1D–1F) validated variant co-segregation with the disease phenotype in the family. Sequencing of missense variant (c.44G>C) in ethnically matched controls was also done to exclude the non-pathogenic nature of the variant. Furthermore, additional 10 family members were Sanger sequenced to remove the occurrence of polymorphic nature of the variant. The substituted Glycine amino acid [p.(Gly15Arg)] located in the SIX5 domain (SD) was conserved across different species (Fig 1G–1I).

### 3.3 Three-dimensional protein modeling

**Fig 2:**
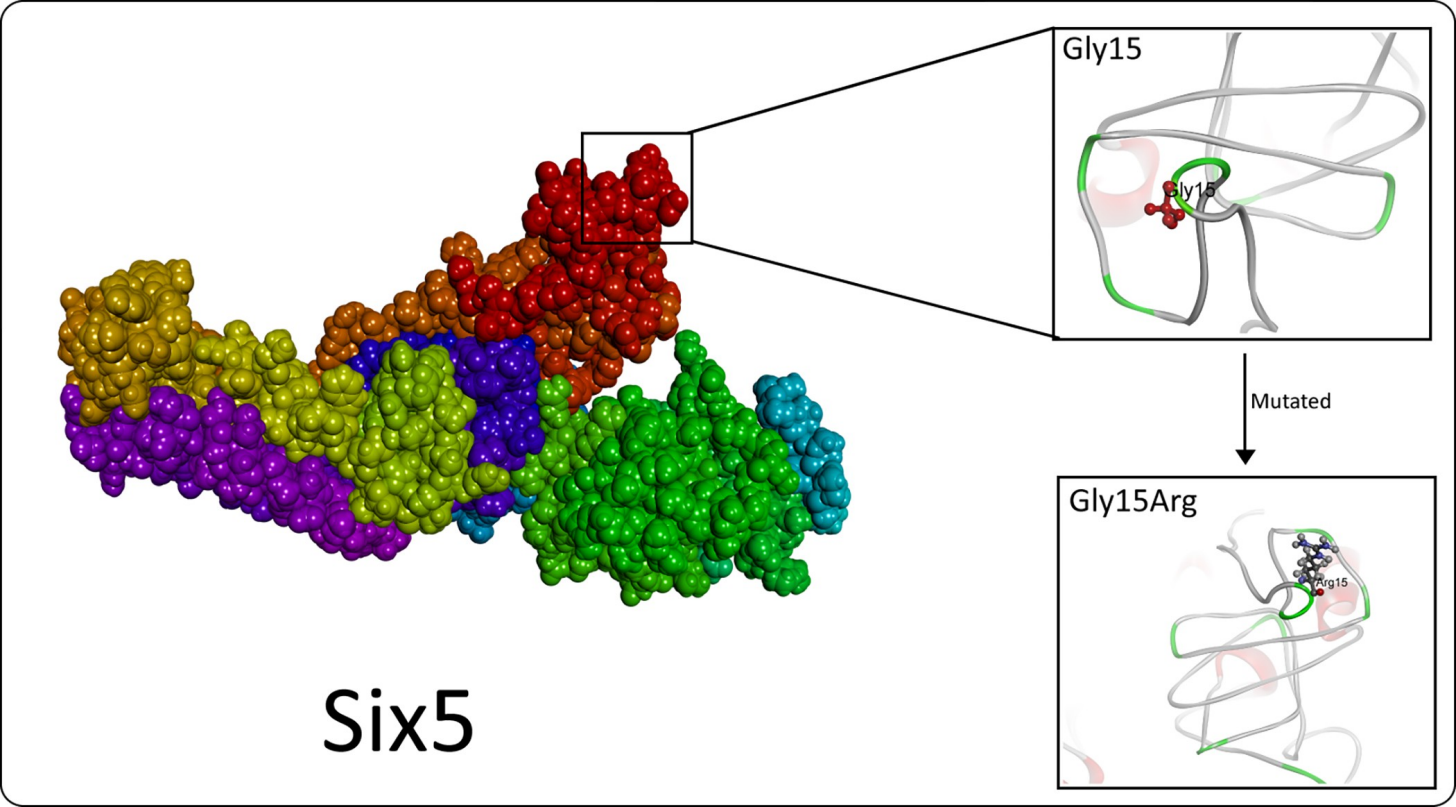
Comparison of wild type and mutated SIX5 protein. The structures of p.Gly15 (wild-type) and p.Arg15 (mutant residues) were adopted by the structures with minimal binding energies. As side chain of residues protrude outside, both residues did not develop any significant intramolecular interaction; however, being in the six domain where EYA family transcriptional factors bind for activation of domain, replaced Arg may change normal interaction with EYA and cause a defect.

**Fig 3:**
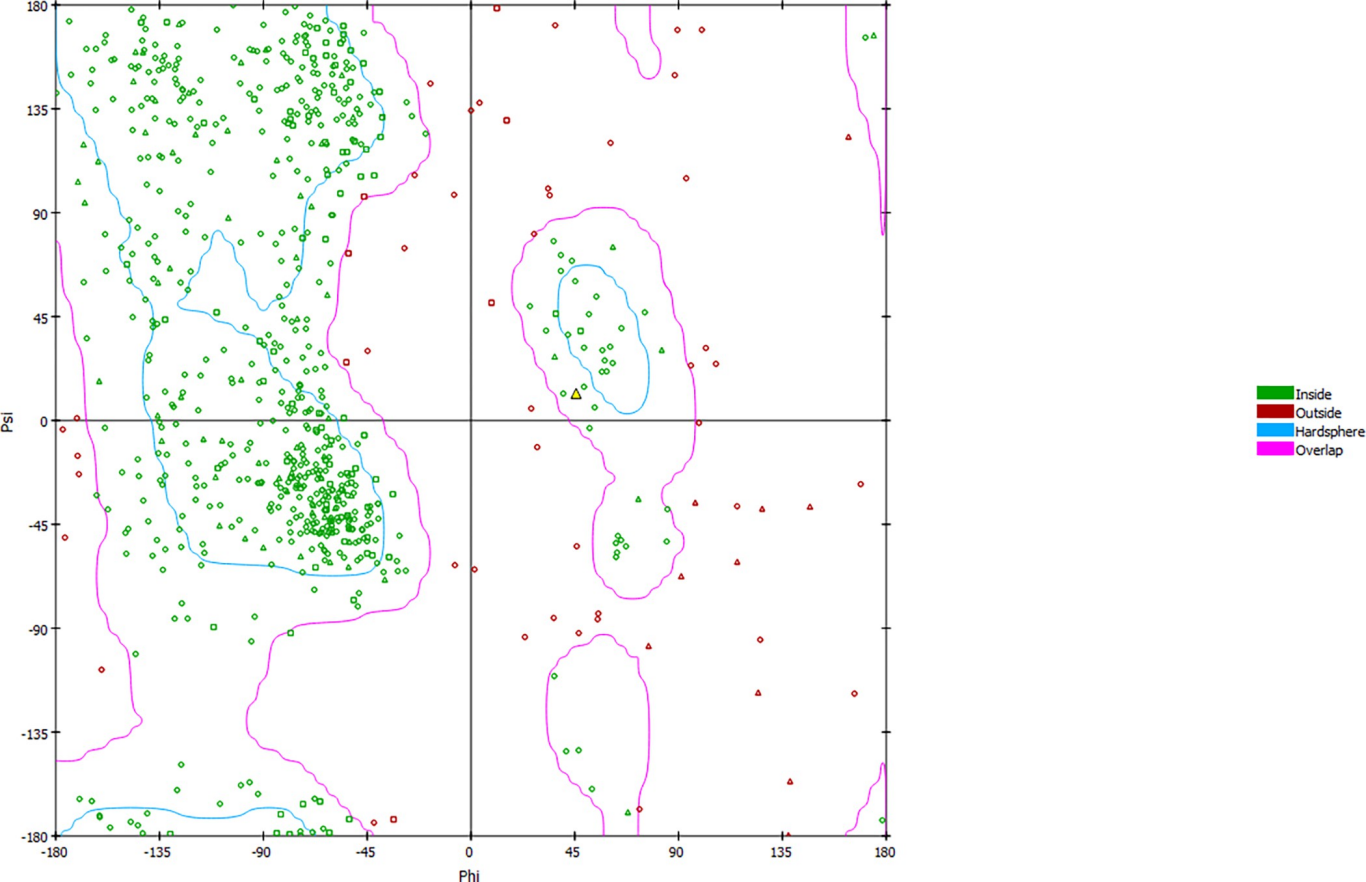
Quality of designed 3D structure of SIX5 protein was evaluated using Ramachandran plot. Overall, 90% residues lie inside allowed regions with a few residues as an outlier, their Phi and Psi angles are not perfectly designed.

Protein review revealed that the identified variant is located in the SIX5 protein’s six domain (Fig 1G and 1H). Moreover, comparative modeling was carried out to determine the structural difference between the wild type SIX5 structure and the mutant SIX5 structure. As the variant [p.(Gly15Arg)] is found in the start of protein where it forms a coil structure, the wild and mutated amino acid (AA) did not develop any interaction with neighboring atoms. The [p.(Gly15Arg)] variant present in the six domains can play a role in deciding DNA-binding specificity as well as mediating protein-protein interactions (EYA1-5), which ultimately activates these (Fig 2). The Ramachandran plot also provided an estimation of the quality of structure. Most of the AA in the 3D model localizes into an estimated region of the Ramachandran plot, as shown in Fig 3.

## 4. Discussion

In this research, we looked at two family members (IV-2 and IV-3) who had prelingual profound dyslexia hearing impairment of a consanguineous family. No vestibular dysfunction or other defects in the affected patients were found, confirming the NSHI patients. WES analysis obtained from HI family members IV-2 and IV-3 showed homozygous variant c.44G>C: [p.(Gly15Arg)] in the *SIX5*. The variant present in the six domain might affect the DNA binding, and protein-protein interaction may cause the hearing defect. Analysis in energy change caused by mutated Arg15 was calculated by the I-Mutant server, which predicted a decrease in stability of the *SIX5* gene (DDG value -0.17 Kcal/mol).

Both recessive (homozygous variant) and dominant (heterozygous variants) in a single gene have been associated with HI in humans. Mutations in the transmembrane channel-like 1 gene (*TMC1*) cause both dominant and recessive HI (DFNA36 and DFNB7/11), respectively (https://omim.org/entry/606706) [20]. Other examples includes, gap junction protein β-3 (*GJB2*) for both DFNA3 (MIM# 601544) and DFNB1 (MIM# 220290) [21, 22], α-tectorin (*TECTA*) for DFNA8/12 and DFNB21 [23], *GJB6* for DFNA3B (MIM# 612643) and DFNB1B (MIM# 612645) [24, 25], *GJB3* for DFNA2B (MIM# 612644) [26, 27], collagen type XI α-2 (COL11A2) DFNA13 and DFNB53 [28], cochlin (COCH) for DFNA9 and DFNB110, respectively [29]. These phenotypic differences show that how different mutations, position specific mutations in a gene and allele combinations (compound heterozygosity) can affect the overall clinical presentation of individuals with HI.

The vertebrate *SIX* genes are similar to the ‘sin oculis’ gene of the Drosophila, which is primarily expressed in the fly’s visual system. SIX gene family members encode proteins that are characterized by a divergent DNA-binding domain and an upstream SIX domain that may aid in determining DNA-binding specificity and mediating protein-protein interactions. Genes of the SIX family play an essential role in insects and vertebrates tissue development and differentiation [30]. SIX family constitutes six genes such as *SIX1*, *SIX2*, *SIX3*, *SIX4*, *SIX5*, and *SIX6* and localized within the GRCh38 locus on chromosome 14q23.1, 2p21, 2p21, 14q23.1, 19q13.32, 14q23.1, respectively [30–35]. Their encoded proteins are widely expressed inside the inner ear and other tissues, including the head, kidneys, heart, skin, and muscles [30]. The presence of SIX genes in different tissues plays an important role in both the linkage and regulation of other genes. The key function of the SIX genes in various diseases is still unclear; however, several studies have shown SIX domain involvement in panic disorders and cancer [36]. These are also involved in the tumorigenesis of liver cancer and can be used as a potential biomarker in predicting the non-small cell lung cancer (NSCLC) patients [36].

SIX5, also known as myotonic dystrophy associated homeodomain protein (DMAHP) is a member of the SIX gene family that codes for proteins with a SIX domain adjacent to a homeodomain [34]. *SIX5* gene provides directions for producing DNA binding proteins and regulating the behavior of other genes. Due to this role, SIX protein is also known as a transcription factor. The SIX5 protein has a molecular weight of 74.5 KDa which interacts with many different proteins, including the EYA1- EYA4, INHA, INHBB, IGBF5, DMPK, DMWD, and DACH1, to control the function of genes essential for average growth [30]. Before birth, these protein interactions are necessary for the normal formation of many tissues, such as the second branchial arch, which triggers tissues in the front and side of the neck, kidneys, and ears. Different studies also identified SIX5 protein expression in the adult brain, eyes, heart, and muscles [34]. DMAHP (SIX5) participates in the pathophysiology of myotonic dystrophy (DM) [37]. DM is a multisystem disease, patient displays progressive muscle wasting with myotonic, cataracts, gonadal atrophy, insulin resistance, heart blockage, and neuropsychiatric impairment. SIX5 dysfunction is associated with the development of adult-onset cataracts, the most common ocular phenotype in DM [38, 39]. Genetic defects are a major cause of NSHI in newborns [40, 41]. Several successful treatments for NSHI have been reported in mice using neonatal gene therapy, neonatal antisense oligonucleotide therapy and embryonic gene therapy [42]. Genetic testing using amniotic fluid is highly useful for prenatal screening of rare genetic diseases. Furthermore, fetal cell-free DNA can be isolated from maternal blood (5th -6th week of gestation). Genetic screening using maternal blood is an attractive option for early genetic diagnosis and treatments [43].

## 5. Conclusion

In conclusion, we investigated the novel biallelic missense variant in the *SIX5* gene causing the severe autosomal recessive HI. This is the first case report of the HI caused by the biallelic variant in the *SIX5* gene. Identifying disease-causing biallelic variants within the *SIX5* gene expands the mutational spectrum and additionally reveals the allelic, clinical, and phenotypic heterogeneity underlying the complex genetic hearing impairment disorder. Furthermore, functional studies and animal models are required for proper genotype-phenotype correlations.

## Supporting information

S1 Table List of genes associated with hearing impairment. (DOCX)

S1 Data (XLSX)
