# Supplementary material for: Identification of homozygous missense variant in SIX5 gene underlying recessive nonsyndromic hearing impairment
Source: PLoS One. 2022 Jun 16;17(6):e0268078. doi: 10.1371/journal.pone.0268078 (PMC9202841; doi:10.1371/journal.pone.0268078)
Supplement: S1 Table — (DOCX) [file pone.0268078.s001.docx]

S1 Table. List of genes associated with hearing impairment

| Order | *Gene name* |
| --- | --- |
| A | *ATP2B2, ACTG1, ALMS1* |
| B | *BSND* |
| C | *CDH23,CLRN1,CLDN14,CCDC50,CEACAM16,COCH,CRYM,COL2A1, CIB2*  *COL11A1,COL9A1,COL9A2,COL4A3,COL4A4,COL4A5,CHD7,CACNA1D* |
| D | *DFNB31, DFNA5,DIABLO,DIAPH1,DSPP, DIAPH3, DLX5* |
| E | *ESRRB,ESPN, EYA1,EYA4, EDNRB,EDN3* |
| F | *FOXI1, FGFR3,FGFR1,FGFR2, FGF3* |
| G | *GJB2,GJB3,GJB6,GIPC3,GRXCR1,GPSM2,GRHL2,GATA3,GLI3,GPR98* |
| H | *HGF, HOXA1, HOXA2* |
| I | *ILDR1, IGF1* |
| K | *KCNJ10, KCNQ4, KCNQ1,KCNE1* |
| L | *LRTOMT, LHFPL5, LOXHD1, LRP2* |
| M | *MYO1A,MYO3A,MYO6,MYO7A,MYO15A,MARVELD2,MSRB3,MIR96,MYH14,MYH9*  *MT-RNR1,MT-TS1, MT-TK,MT-TE,MT-TL1, MITF* |
| N | *NDP,NLRP3* |
| O | *OTOF, OTOG, OTOA, OPA1* |
| P | *PCDH15,PTPRQ,POU4F3,POU3F4,PRPS1,PDSS1,PHEX,PAX2,PAX3,PRRX1, PDZD7* |
| R | *RDX* |
| S | *SLC17A8,SLC26A4,SLC26A5,STRC,SERPINB6,SIX1,SIX5,SMPX,SERAC1,SOX9,SLC19A2*  *SNAI2, SOX10, SOBP, SEMA3E,SMAD4, SLC4A11* |
| T | *TMIE,TMC1,TMPRSS3,TECTA,TRIOBP,TPRN,TJP2,TNFRSF11B,TCOF1,TIMM8A* |
| U | *USH1C, USH1G,USH2A* |
| W | *WFS1* |
